# eNose analysis of volatile chemicals from dogs naturally infected with *Leishmania infantum* in Brazil

**DOI:** 10.1371/journal.pntd.0007599

**Published:** 2019-08-06

**Authors:** Monica E. Staniek, Luigi Sedda, Tim D. Gibson, Cristian F. de Souza, Erika M. Costa, Rod J. Dillon, James G. C. Hamilton

**Affiliations:** 1 Division of Biomedical and Life Sciences, Faculty of Health and Medicine, Lancaster University, Lancashire, United Kingdom; 2 Centre for Health Informatics Computation and Statistics, Lancaster Medical School, Faculty of Health and Medicine, Lancaster University, Lancashire, United Kingdom; 3 RoboScientific Ltd., Espace North, Littleport, Cambridgeshire; 4 Fiotec (Rio de Janeiro), Avenida Brasil, Manguinhos, Rio de Janeiro, Brazil; 5 Laboratório de Pesquisa em Leishmaniose, Instituto Oswaldo Cruz, Rio de Janeiro, Brazil; Universidade do Estado do Rio de Janeiro, BRAZIL

## Abstract

**Background:**

Visceral leishmaniasis (VL) in Brazil is a neglected, vector-borne, tropical parasitic disease that is responsible for several thousand human deaths every year. The transmission route involves sand flies becoming infected after feeding on infected reservoir host, mainly dogs, and then transmitting the *Leishmania infantum* parasites while feeding on humans. A major component of the VL control effort is the identification and euthanasia of infected dogs to remove them as a source of infection. A rapid, non-invasive, point-of-care device able to differentiate between the odours of infected and uninfected dogs may contribute towards the accurate diagnosis of canine VL.

**Methodology/Principal findings:**

We analysed the headspace volatile chemicals from the hair of two groups of dogs collected in 2017 and 2018 using a bench-top eNose volatile organic chemical analyser. The dogs were categorised as infected or uninfected by PCR analysis of blood samples taken by venepuncture and the number of parasites per ml of blood was calculated for each dog by qPCR analysis. We demonstrated using a robust clustering analysis that the eNose data could be discriminated into infected and uninfected categories with specificity >94% and sensitivity >97%. The eNose device and data analysis were sufficiently sensitive to be able to identify infected dogs even when the *Leishmania* population in the circulating blood was very low.

**Conclusions/Significance:**

The study illustrates the potential of the eNose to rapidly and accurately identify dogs infected with *Le*. *infantum*. Future improvements to eNose analyser sensor sensitivity, sampling methodology and portability suggest that this approach could significantly improve the diagnosis of VL infected dogs in Brazil with additional potential for effective diagnosis of VL in humans as well as for the diagnosis of other parasitic diseases.

## Author summary

Visceral leishmaniasis (VL) is an insect transmitted, tropical parasitic disease and in Brazil it causes thousands of human deaths every year. Domestic dogs can also be infected, and they are a risk factor for people. The Brazilian Ministry of Health tries to control the disease in 3 ways; first by reducing the population of insects that can carry the disease, second by using therapeutic drugs to treat the disease in humans, and third by identifying and euthanising infected dogs. However, despite these efforts the burden of VL has doubled since 2010 and a significant contributing factor is the lack of a rapid and accurate pathway for diagnosing dogs. In this study we have shown that an eNose can differentiate between the smell of VL infected and uninfected dogs. The analysis was highly sensitive i.e. if the dog was infected, the eNose would detect it in > 97% of the cases and it was highly specific i.e. if the dog was uninfected eNose detect it in >94% of the cases. The outcome was not dependant on the numbers of parasites or the clinical status of the dog. The results suggest that eNose analysis could be used to identify VL infected dogs with improved the speed and accuracy compared to current methods.

## Introduction

Visceral leishmaniasis (VL) is a neglected tropical disease caused by protist parasites belonging to the genus *Leishmania*. Globally over 350 million people are at risk of infection with an estimated 200–400 thousand cases annually and an estimated 10% fatality rate. Ninety percent of all reported VL cases occur in only six countries including Brazil[1, 2].

In Brazil, transmission of *Leishmania (Leishmania) infantum* (Kinetoplastida: Trypanosomatidae) occurs between domestic dogs *Canis familiaris* (Carnivora: Canidae) (the reservoir host) and from dogs to humans when an infected female sand fly vector *Lutzomyia longipalpis* (Diptera: Psychodidae) takes a blood meal.

Despite substantial efforts by the Brazilian Ministry of Health (MoH) the burden of VL in Brazil more than doubled between 1990 and 2016[3]. The increase is probably due to the spread of the vector into urban areas as a result of human migration into cities[4] and the expansion of the range of the vector into new areas because of environmental degradation[5–7]. Given the spread of the disease and increase in cases it is also likely that current VL control measures are inadequate[8].

The control of VL in Brazil has three main components. Insecticides are applied in houses and animal sheds to lower the vector population density and reduce vector-human contact. Secondly, diagnosis and treatment of human cases to prevent severe forms of the disease and death. Finally, the identification and euthanasia of seropositive canine cases to decrease the sources of infection for the vector[9, 10].

Modelling predicts that the dog-culling program in Brazil should be effective in areas of low, medium but not high *Leishmania* transmission[11]. However, the practice is controversial and despite the euthanasia of thousands of canines with suspected and confirmed infection each year the program has been unsuccessful[10, 12]. There are a number of possible explanations for this situation A). Shortage of qualified professionals caused by financial constraints leading to delays in collections, performance of routine diagnostic tests and subsequent removal of seropositive dogs. B). Failure to identify and remove the high proportion of asymptomatic animals C). Refusal of dog owners to comply with surveillance measures. D). The high rate of dog replacement with young immunologically naïve dogs. E). Lack of an accurate point-of-care diagnostic test[13].

Identification of dogs infected with canine VL (CVL) follows a two stage serodiagnostic protocol recommended by the Brazilian MoH. Initial screening using the Dual-Path Platform (DPP CVL) immunochromatography diagnostic test is followed by a laboratory-based ELISA (EIE CVL) confirmatory test. Overall the 2-step protocol was reported to have a 73% sensitivity and 98% specificity however the relatively low sensitivity indicates the maintenance of false-negative dogs in endemic areas which represents a public health concern[14].

The DPP CVL test has also been assessed several times since it was introduced and most recently it has been shown that overall it has 86% sensitivity and 94% specificity[14] or 89% sensitivity and 70% specificity[15].

The concept of volatile organic compounds (VOCs) as diagnostic aids to signal a disease is well established and since antiquity, many physicians have used odours associated with disease to help diagnose their patients[16]. Modern analytical techniques such as single ion flow tube mass spectrometry (SIFT-MS) and chemi-resistive sensors have taken the concept to the point of widespread clinical application. Volatile markers from human breath can be used to identify a variety of disease states e.g. inflammatory bowel disease, chronic liver disease, diabetes, *Pseudomonas aeruginosa* infection and adenocarcinomas[17, 18]. A recent study has shown that the use of VOCs is sufficiently robust to discriminate between 14 cancerous and other disease states[19].

Parasite infections of humans and animals also alter the odour of the host animal. The odours of golden hamsters infected with *Le*. *infantum* are more attractive to female sand flies than the odours of uninfected hamsters[20, 21]. The odour obtained from the hair of dogs infected with *Le*. *infantum* in Brazil was found to be significantly different to the odour of uninfected dogs. These odour differences which were detected by coupled gas chromatography-mass spectrometry (GC/MS) and multivariate statistical analysis indicated the increased presence of a small number of primarily low molecular weight aldehydes (octanal, nonanal), alkanes (undecane, heptadecane) and 2-ethylhexyl-salicylate[22, 23]. More recently, odours were also implicated in children infected with the infectious gametocyte stage of the malaria parasite *Plasmodium falciparum were* found to be more attractive to the mosquito vector *Anopheles gambiae*[24]. This phenomenon occurred even when the gametocytemia was very low and was associated with changes in aldehyde concentration of the foot odours of the infected children[25, 26].

GC/MS analysis is a useful research tool but its use as a widely available diagnostic tool is unrealistic because of significant costs associated with the infrastructure and personnel costs. An alternative means of detecting the odour change associated with parasitaemia is required that would fulfil the majority of the World Health Organisation ASSURED criteria; affordable, sensitive, specific, user-friendly, rapid and robust, equipment free and deliverable to end-users[27] for all new point-of-care diagnostics tools. VOC analysers (eNoses) may fulfil WHO criteria, they can detect differences in the odours from sputum of tuberculosis (TB) infected and TB uninfected patients with sensitivity, specificity and accuracy of around 70%[28]. The aim of the present study was to determine if the odour of dogs naturally infected with *Le*. *infantum* could be detected with high sensitivity and specificity using a commercially available VOC analyser.

## Methods

### Study site

Governador Valadares (18°51′S, 41°56′W) (Minas Gerais State, Brazil), located in the valley of the Rio Doce 320 km northeast of Belo Horizonte is a city of approximately 280,000 people. The climate is temperate, characterised by dry winters and hot, wet summers[29]. Studies in Governador Valadares in 2013 found that an average of 30% of dogs from 16,529 samples taken from 35 urban and rural districts were seropositive for canine visceral leishmaniasis (CVL)[30]. From 2008 until 2017, 194 human VL cases were recorded in Governador Valadares with a fatality rate of 15.5%[31].

### Ethics

Dog blood and hair samples were taken from dogs that were also microchipped with the informed consent of their owners. Ethical approval was obtained from the Comissão de Ética no Uso de Animais (CEUA), Instituto Oswaldo Cruz (licence L-027/2017) in Brazil and Lancaster University Animal Welfare and Ethics Review Board (AWERB) in the UK. The CEUA approval complies with the provisions of Brazilian Law 11794/08, which provides for the scientific use of animals, including the principles of Brazilian Society of Science in Laboratory Animals (SBCAL). The AWERB approval complies with the UK Home Office guidelines of the Animals in Science Regulation Unit (ASRU) and in compliance with the Animals (Scientific Procedures) Act (ASPA) 1986 (amended 2012) regulations and was consistent with UK Animal Welfare Act 2006.

### Dog recruitment

A 2 year cohort study in the Altinópolos district of Governador Valadares was initiated in August 2017 by initial recruitment and sampling of 185 dogs. The area was chosen because of the high prevalence of CVL (average incidence 33.8%)[30] and the large population of household-owned dogs (ca. 2000) (Centro de Controle de Zoonoses (CCZ) survey) located there. The dogs were microchipped to aid their identification. Inclusion criteria: dogs aged ≥ 3 months, dogs without previous clinical assessment or laboratorial diagnosis for CVL. Exclusion criteria: pregnant/lactating bitches; aggressive dogs; stray dogs. In April 2018 149 dogs were sampled, this number included 133 dogs that were resampled from the 2017 cohort and an additional 16 from CCZ which had been collected in the same area and at the same time as our sample collections.

Between 5ml and 10ml of peripheral blood was collected in 10ml K2 EDTA-coated tubes (BD Vacutainer, UK) via cephalic or jugular venepuncture by a qualified vet in 2017 and by a CCZ qualified phlebotomist in 2018. Samples were placed in containers marked with the microchip bar code to aid subsequent tracking and identification. Blood samples were stored in a cool box with a freezer pack before being transferred to a fridge (4°C) prior to processing.

Hair samples were obtained by cutting the dorsal hair close to the skin using surgical scissors that had been washed with hexane prior to the collection of each sample by members of the LU research team. A minimum of 2g of hair was collected from each dog. All hair samples were placed in individual foil bags (110mm x 185mm; Polypouch UK Ltd, Watford, England) heat sealed and stored at 4°C prior to analysis.

All dogs were assessed for clinical signs of *Leishmania* infection by veterinarians and CVL control specialists at CCZ. The animals were classified according to the presence of clinical signs which were recorded for each dog. The main signs of CVL considered were onychogryphosis, ophthalmologic abnormalities, adenitis, cachexia, hepatosplenomegaly, alopecia, crusted ulcers and lesions; dogs were classified as asymptomatic (the absence of clinical signs), oligosymptomatic (the presence of one to three clinical signs), or symptomatic (the presence of more than three clinical signs[32].

### Molecular diagnosis of dogs

#### DNA extraction

Collected blood samples were centrifuged at 2500 x g for 10 minutes at room temperature and the top layer of buffy coat removed, placed in 1.5ml Eppendorf tubes and stored at -20°C until DNA extraction. The DNA was extracted from 200μl of buffy coat samples using the QIAamp DNA Blood Mini Kit following the manufacturer’s instructions. Cell lysis was mediated using protein kinase with a final elution volume of 50μl.

#### Qualitative detection of Leishmania DNA

Conventional PCR was initially used to ascertain which blood samples were positive for *Leishmania infantum*. Although the sensitivity of PCR is not 100% and is not considered to be as accurate as the direct parasitological assessment of lymph node or bone marrow aspirates, it is highly sensitive, rapid, requires minimal facilities, avoids potential dog-odour contamination issues and is less distressing for the dogs.

Following primer optimization, extracted DNA from canine blood obtained during August 2017 and April 2018 were tested using Primer pair MaryF (5’–CTT TTC TGG TCC TCC GGG TAG G– 3’), and MaryR (5’- CCA CCC GGC CCT ATT TTA CAC CAA– 3’ [33]. The reactions were performed in a final volume of 25μl containing 0.5μl DNA template (100ng μl-1), 12.5μl Mastermix (dH_2_O, Buffer 5x, MyTaq redmix polymerase, dNTP’s) and 10μM of each primer. The PCR amplifications were performed in a TECHNE Prime Thermal Cycler (Cole-Palmer Ltd., Staffordshire, UK) using the following conditions: 95°C for 5mins and 30 cycles of 95°C for 30sec, 57°C for 30sec and 72°C for 60sec, followed by 72°C for 10min.

The PCR products were analysed by gel electrophoresis using 2% agarose gels run at 90V for 1hr 30 minutes and visualized under UV light following the addition of 6.5μl of 10,000x SYBR Safe (Thermo Fisher Scientific, UK) to each gel. Samples were run 3 times and dogs were considered to be infected if 2 or 3 out of the 3 replicates were positive.

Canine beta globin house-keeping gene was used to monitor the performance of the amplification and check for DNA degradation as the samples were transported from Brazil to the UK. Amplification of the constitutive canine globin gene was performed using the primers: ‘5—CAA CTT CAT CCA CGT TCA CC– 3’ and ‘5—ACA CAA CTG TGT TCA CTA GC– 3’[34]. Positive control was leishmania culture DNA at a 10^6^ parasites ml^-1^ concentration. Negative control was obtained by performing DNA extraction on 200μl of water instead of buffy coat in Brazil under the same conditions as the blood.

#### Quantitative detection of Leishmania DNA

A real-time quantitative PCR (qPCR) for detection and quantification of *Le*. *infantum* DNA in positive dog samples from both sampling occasions (August 2017 and April 2018) was performed using MaryF/R primers.

The qPCR amplifications were performed on a Bio-Rad C1000 Thermal Cycler with each reaction consisting of a final volume of 13.0μl; 12.0μL of PCR mix plus 1μL of DNA (approximately 75–100 ng/μl per reaction). The qPCR mix was composed of 6.25 μL 2x QuantiNova SYBR Green PCR Master Mix, 0.5 μL of each primer (MaryF/R, corresponding to 10 mmol) and 4.75 μL of water[35].

The amplification was performed in triplicate[36] at 94°C for 10 min, followed by 40 cycles at 94°C for 30 sec, 60°C for 20 sec and 72°C for 20 sec. At the end of each run, a melt curve analysis was performed from 55°C to 95°C in order to identify the formation of non-specific products as well as primer dimers. A standard curve was established using extracted *Le*. *infantum* DNA; 1:10 serial dilutions, ranging from 10,000 to 0.01 parasites per ml and used to quantify the number of parasites in the dog blood samples.

### VOC analysis

Initial VOC analysis was carried out on all (n = 11) of the infected dog hair samples and a sub-set of the uninfected dog hair samples (n = 44) collected in 2017. The choice of 44 uninfected dog hair samples (4 matched uninfected dog hair samples for each infected dog hair sample) was obtained by a power analysis to optimise the control size. The uninfected dogs were selected from groups of dogs matched by shared characteristics (age, sex and whether or not treatment for ectoparasites was received) with infected dogs (Table 1). Subsequently the VOC analysis was carried out on all the infected dog hair samples (n = 44, including 10 CCZ infected dogs) and all of the uninfected dog hair samples (n = 105, including 6 CCZ uninfected dogs) collected in 2018. The number of uninfected dog hair did not exceed the “4 uninfected for each infected dog” rule set above, and for this reason all the dogs were used.

**Table 1:** *Leishmania infantum* infection status of dogs sampled in Governador Valadares, Minas Gerais.

Month of sample collection	Source of dogs	Number of dogs sampled	Leishmania positive dogs (PCR)		
			symptomatic	oligosymptomatic	asymptomatic
Aug-17	Altinópolis	185	0	3	8
Apr-18	Altinópolis	133	3	7	24
Apr-18	CCZ GV	16	6	2	2

Dogs from Altinópolis, Governador Valadares sampled in August 2017 (n = 185) and April 2018 (n = 133). An additional 16 dogs, considered positive by CCZ, were sampled in April 2018. PCR experiments were performed in triplicate with both positive and negative dogs identified.

A VOC analyser (Model 307, RoboScientific Ltd, Leeds, UK) with 11 functioning semi-conducting polymer sensors was used for the analysis. Each sensor has 2 outputs (positive and negative) giving a total of 22 responses. Two calibration points were automatically set by the sensor unit; the first was the baseline obtained when carbon-filtered air was passed over the sensor at a flow rate of 200ml min^-1^ which was automatically adjusted to zero on the Y-axis scale, and the second was a reference point obtained from sampling the head space of 5ml of a liquid water control in a plastic vial.

The chemical sensors were thin films of semi-conducting polymers deposited onto interdigitated gold structures on a silicon substrate. We used 12 different sensor types chosen from a group of polymers that included polyaniline, polythiophene and polypyrrole. Each sensor had semi-selectivity to a different group of volatile chemicals; aldehydes, alcohols, amines, organic acids and ketones etc. In this way a digital fingerprint of the VOC mixtures emanating from the samples was generated. Two similar sensor arrays were used in the study, the second array (used for the 2018 analysis) was a derivation of the first with 50% of the sensors being identical to the first array.

The interaction of the mixtures of VOCs in the samples with the semi-conducting polymer surfaces produced a change in electrical properties (e.g. voltage and resistance) over time. This change was measured, recorded and simultaneously displayed on the VOC analyser data logger screen for each sensor. Four parameters were used from each sensor response; the divergence from the baseline (maximum response), the integrated area under each response curve, absorbance and desorbance. Therefore, the total number of VOC measurements produced for each sample were 88 (11 sensors x 4 parameters and 2 outputs–positive or negative). The sampling profile was set at 2 seconds baseline, 7 seconds of absorption, a 1 second pause, 5 seconds desorption and 12 seconds flush to bring the sensors back to baseline.

Water (DD;10μl) was injected into each foil bag containing the dog hair samples with a Hamilton syringe and inflated with 140ml of laboratory air using a diaphragm pump. The samples were then incubated at 50°C for 15 minutes in an oven, then allowed to cool to room temperature for 5 minutes prior to head space analysis.

For the analysis each foil bag (containing the dog hair + water) was sampled by insertion of an 18-gauge needle connected to a PTFE tube through the sidewall of the bag with the tip placed into the head space of each bag. This was connected to the sample port of the VOC analyser and the head space sample was therefore passed over the 12 sensor surfaces. The original flow rate for the sampling was 200 ml min^-1^. The headspace of each foil bag was sampled 4 times. The first sample was disregarded as potentially it could contain volatile carryover from the previous sample and thus, we retained the data from the next 3 samples for analysis. The individual dog hair samples in each experiment were tested randomly with each sample used once only.

### Data analysis

To test the ability of the VOC analyser to differentiate between the odours of infected and uninfected dogs, we employed mclust[37], a model-based clustering and classification algorithm (R-CRAN statistical software[38]. This was applied to the known data classes (infected or uninfected dogs). The initial analysis indicated that the model was overfitted, therefore we identified the infected and uninfected dog sub-classes (unsupervised clustering) and the analysis was repeated [39]. The robustness of the classification was evaluated by out-of-sample cross validation (CV) while the within-group homogeneity of the overfitting models was evaluated by a novel algorithm developed by the authors and termed confounder cross validation (CCV). Finally, the importance of each variable produced by the VOC analyser in discriminating between the infected and uninfected sub-classes was assessed by variable permutation analysis. A more detailed explanation of the rationale for this analysis approach is provided in the S1 Material and a more extensive description of the algorithms is provided in[40].

The VOC analysis dataset contained data from:

- Infected and uninfected dog hair collected in 2017.
- Infected and uninfected dog odour collected in 2018 including samples collected from CCZ dogs.

Three replicate VOC analyser readings were obtained for each dog odour sample. These replicates were considered to be independent, i.e. the three VOC replicates for each dog were considered as coming from three different dogs (a common procedure for repeated data in clustering analyses).

The analysis aimed to identify any significant differences in the VOC analyser variables (used to obtain the means and covariances of the infected and uninfected classes and/or sub-classes) of infected and uninfected dogs so as to be able to accurately predict the infection state of newly sampled dogs. Initially, the data was evaluated to determine 1. if infected and uninfected dogs in both 2017 and 2018 could be statistically separated and 2. if the uninfected dogs in 2017 were statistically separate from uninfected dogs in 2018.

As described above, to take account of overfitting [41], we reclassified the infected and uninfected classes into sub-classes using the mclust function (mclust package). The optimal inferential method and number of subclasses for infected and uninfected classes was obtained by Bayesian information criterion (BIC) (S1 Material), bootstrapping and the likelihood ratio test (function mclustBootstrapLRT (mclust package).

#### Importance of variables: Cross-validation (CV) and confounder cross-validation (CCV) analyses

Once the best model (number of subclasses and model components) had been found, we tested for the importance of the variables in clustering by permutation analysis; while the predictive capacity of the model by using “leave-one-out” cross validation (CV); and finally, the capacity of the model to recognise sample confounders by developing a technique named confounder cross-validation (CCV) (a test to evaluate the statistical homogeneity of the class). For the latter 10% of the data from the infected class were placed in the uninfected class and vice versa (leaving the remaining 90% in their correct class for training in both cases). These analyses were done by compiling algorithms that included some of the MCLUST components (Mclust, MclustDA, predict) and permutation functions. Additional information is provided in the S1 Material.

## Results

### Molecular diagnosis of dogs

#### Qualitative detection of Leishmania DNA

**Fig 1:**
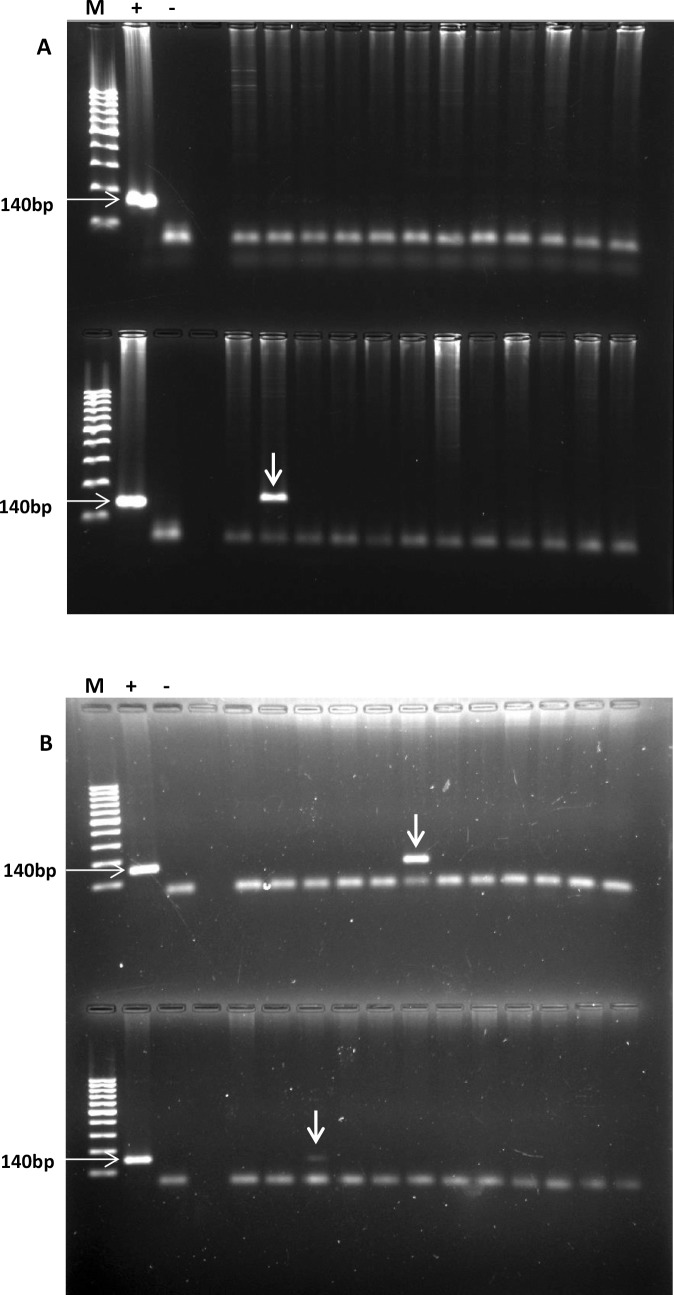
Preliminary detection of *Leishmania infantum* in dog blood samples. SYBR safe-stained 2% agarose gels showing the results of electrophoresis of 24 random PCR products from A; August 2017 dogs and B; April 2018 dogs, used to identify the presence of *Leishmania* in dog blood. Positive dogs are indicated by a 140bp band visible in line with the PCR product for the *Leishmania* positive control. M, molecular weight marker (100bp DNA ladder); “+” = positive control; “-” = negative control.

PCR revealed that 11/185 (6%) dogs were positive for *Le*. *infantum* infection in August 2017 and 34/133 (26%) in April 2018 (Table 1) representing a 20 percent increase in infection rate over the 8-month period between sampling points. The typical PCR results showed a band at 140bp of varying intensities representing a semi-quantitative indication of parasite presence in individual samples (Fig 1).

In 2017, 3 out of the 11 positive dogs presented as oligosymptomatic and 8 were asymptomatic. In 2018, 3 dogs were symptomatic, 7 were oligosymptomatic and 24 were asymptomatic (Table 1). Of the 174 uninfected 2017 dogs 42 were lost to follow-up in 2018. There were 7 oligosymptomatic dogs and 3 had become symptomatic in the remaining 133 dogs. In the 2017 cohort 55% of the infected dogs had 3 out of 3 positive PCR results and 45% had 2 out of 3 positive PCR results. In the 2018 field collected cohort 47% had 3/3 + PCR results and 53% had 2/3 + PCR results. In the 2018 CCZ collected cohort 80% had 3/3 + PCR results and 20% had 2/3 + PCR results. Of the 11 positive dogs found in 2017 only 1 (dog 105) was resampled in 2018. The fate of all the loss to follow-up dogs (n = 52) was recorded by CCZ and in common with other surveys[42] the dogs had mostly either; died (n = 20; 3 through non-illness related issues) or escaped, became lost or stolen (n = 4), their owners moved (n = 13) or refused further testing (n = 7).

The evaluation indicated that the most frequently occurring clinical signs were skin lesions including dermatitis (18% 2017; 27% 2018) and ulcerative lesions (0% 2017; 25% 2018), long nails (9% 2017; 25% 2018) and signs of conjunctivitis (18% 2017; 14% 2018).

PCR diagnosis of the 16 CCZ dogs, sampled in April 2018, that were assumed to be VL infected, indicated that 10 (63%) were positive and the remaining 6 cases were not infected.

#### Quantitative detection of Leishmania DNA

**Fig 2:**
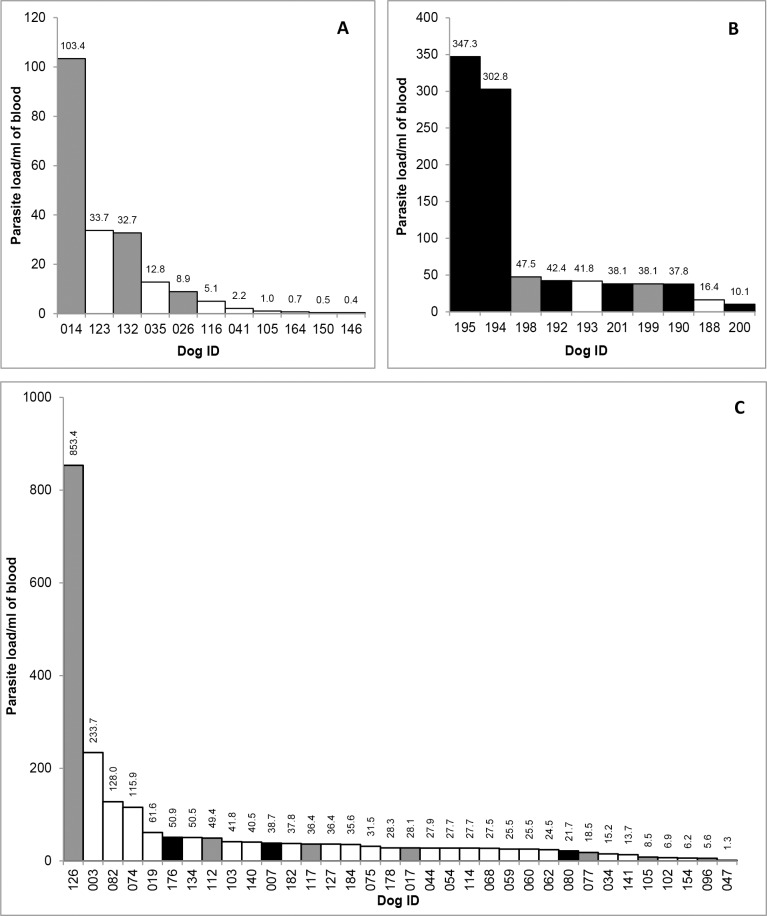
Quantitative estimation of *Leishmania infantum* in blood samples from infected dogs. kDNA qPCR assay showing the quantification of all positive samples from A; August 2017, B; CCZ (April 2018) and C; in the field (April 2018). All positive samples previously determined by conventional PCR were qualitatively analysed by qPCR to determine parasite loads of each positive dog. Range of parasite load; 0.4 parasites mL^1^ to 853 parasites mL^-1^. White bars; asymptomatic dogs, grey bars; oligosymptomatic dogs and black bars; symptomatic dogs. Parasite load is per mL of blood. For clarity parasite load is given for all dogs in 2A and 2B but is excluded from dogs with similar loads in 2C. Dog identification number is given on the X-axis.

The kDNA qPCR assay showed that parasite loads ranged from 0.4 to 103 parasites ml^-1^ in 2017 and from 1 to 850 parasite ml^-1^ in 2018 (both field and CCZ collected) (Fig 2).

This large variation in parasitic load over the study period can be observed through analysis of the median values which ranged from 5.06 parasites/ml (dog #116) in 2017 to 28.32 parasites/ml (dog #178) in 2018. Comparisons of parasitic load among the samples revealed that dog #126 in 2018 (853 parasites ml^-1^) exhibited the highest degree of parasitism with dog #146 in 2017 (0.4 parasites ml^-1^) exhibiting the lowest.

The average value of CT (ΔCT) obtained for dog #126 in 2018 (highest degree of parasitism) and for dog #146 in 2017 (lowest degree of parasitism) were the following: dog #126; 20.43 and dog #146; 30.45. A lower CT value correlates with a higher parasitic load per ml of blood.

### Data analysis

The best model for the analysis of all infected and uninfected dog classes was EEE apart from the uninfected 2017 dogs which was VVI [40]. The EEE model assumes ellipsoidal covariances and equal shape, volume and orientation for all the classes. The VVI model assumes diagonal covariances with orientation parallel to the coordinate axes with variable shape and volume for all the classes [43]. Between 14 models x 1 to 9 classes were assessed (i.e. 126 mixture models) [44] (S1 Table (2017 data) and S2 Table (2018 data)). Clustering analysis of 2017 dogs identified 1 class for uninfected dogs and 3 classes for infected dogs and for the 2018 dogs 2 classes for uninfected dogs and 6 classes for infected dogs were identified.

Confusion matrices of the separation obtained from the training set of uninfected vs infected dogs in 2017 and uninfected vs infected dogs in 2018 without sub-classes are given in Table 2A and 2C respectively and with sub-classes in Table 2B and 2D respectively below.

**Table 2:** Confusion matrices for Gaussian mixture model EDDA classification.

A. 2017: 1 uninfected and 1 infected dog class.				
observed\predicted	uninfected	infected	specificity	sensitivity
uninfected	111	1	0.99 (0.95,0.99)	
infected	3	28		0.90 (0.75,0.96)
training error:	0.028			
B. 2017. VVI model: 1 uninfected and 3 infected dog classes.				
uninfected	112	0	1 (0.96,1)	
infected	0	31		1 (0.88,1)
training error:	0			
C. 2018: 1 uninfected and 1 infected dog class.				
uninfected	280	34	0.89 (0.85,0.92)	
infected	0	132		1.00 (0.97,1)
training error	0.076			
D. 2018. EEE model: 2 uninfected and 6 infected dog classes.				
uninfected	298	16	0.94 (0.91,0.96)	
infected	3	129		0.97 (0.93,0.99
training error	0.042			

Training error is the average error, i.e. the ratio between correctly predicted class members and the total number of records e.g. in 1A above (111+28)/(111+28+1+3). Specificity and sensitivity 95% confidence intervals (based on binomial probabilities) are reported in brackets.

These data show that in both years the infected dog odours were significantly different from the uninfected dog odours. In 2017 uninfected dogs were discriminated with 96% specificity and 97% sensitivity, that was improved to 100% for both metrics when the data was divided in sub-classes. The overall training error was reduced (from 2.8% to 0%) when the 2017 data was divided in sub-classes.

In 2018 uninfected and infected dogs were discriminated with 89% specificity and 100% sensitivity and that was improved to 94% specificity and 97% sensitivity when the data was divided in sub-classes. The overall training error was reduced (from 7.6% to 4.2%) when the 2018 data was divided in sub-classes.

#### Cross-validation (CV) and confounder cross-validation (CCV) analysis

When considering only the infected and uninfected classes, the CV analysis returned a reduced sensitivity of 50% and specificity of 84% for 2017 dogs, and 48% sensitivity and 96% specificity for 2018 dogs (Table 3A and 3B first line and first two columns) indicating a reduced capacity to estimate true positives compared to the training set (as reported in Table 2) due to overfitting. The CCV analysis suggested high heterogeneity of the infected and uninfected classes since the model is unable to identify the false positive and false negatives in the training groups. However, when the analyses were repeated on the EDDA models with sub-classes, both cross validation (CV) and confounder cross validation (CCV) calculations of sensitivity and specificity improved substantially (Table 3A and 3B second line). Thus, by identifying sub-classes for infected and uninfected dogs it was possible to obtain a better delineation of the multivariate space with improved predictivity capacity (CV analysis) and recognition of false positive and false negative in the two main macro-classes (infected and uninfected) of the training sets (increased homogeneity into sub-classes; CCV analysis).

**Table 3:** Comparison of sensitivity and specificity after CV and CCV analysis.

**A**	2017 dog samples			
Model	CV sensitivity	CV specificity	CCV sensitivity	CCV specificity
2 classes	0.50 (0.40,0.59)	0.84 (0.75,0.89)	0.11 (0.09,0.13)	0.60 (0.56,0.62)
4 classes	0.75 (0.65,0.82)	0.80 (0.71,0.86)	0.60 (0.56,0.62)	0.70 (0.67,0.72)
2 classes = 1 uninfected + 1 infected class			4 classes = 1 uninfected + 3 infected classes		
**B**	2018 dog samples				
2 classes	0.48 (0.38,0.57)	0.96 (0.90,0.98)	0.18 (0.15,0.20)	0.67 (0.64,0.69)
8 classes	0.93 (0.86,0.96)	0.92 (0.85,0.95)	0.74 (0.71,0.76)	0.84 (0.81,0.86)
2 classes = 1 uninfected + 1 infected			8 classes = 2 uninfected + 6 infected	

Specificity and sensitivity 95% confidence intervals (based on binomial probabilities) are reported in brackets. CV- cross validation; CCV- confounder cross validation.

The eNose variables important in the clustering are shown in S3 Table (2017 data) and S4 Table (2018 data). A 0.99 P-value indicates that in 99% of the permutations the number of optimal clusters changed, indicating a strong influence of the variable in the final clustering.

## Discussion

The results presented in this study show that by combining VOC (eNose) data with robust clustering analysis we can identify dogs infected with *Le*. *infantum* by analysis of their odour with very high sensitivity and specificity, regardless of parasite load or the presentation of clinical symptoms. We observed this outcome in two data sets from dog hair samples collected in 2017 (99% [0.95,0.99] specificity and 90% [0.75,0.96] sensitivity) and in 2018 (89% [0.85,0.92] specificity and 100% 0.97,1] sensitivity). When the small size of both data sets (2017, 55 dog hair samples: 2018, 149 dog hair samples) and consequent potential for overfitting was accounted for by improving the mixture of the models, both sensitivity and specificity increased (2017; 100% [0.96,1] specificity, 100% [0.88,1] sensitivity: 2018; 94% [0.91,0.96] specificity, 97% [0.93,0.99] sensitivity).

The robustness of the models was further tested by cross-validation and an novel approach which we have termed confounder cross validation analyses. The model prediction was poor when we used 2 classes (infected and uninfected). However, when we accounted for the heterogeneity within each of these classes and subdivided them into either 4 subclasses (2017 data) or 8 subclasses (2018 data) the sensitivity and specificity and their confidence intervals improved substantially. In both cases the models accurately placed dog odours in the correct infected or uninfected class with a high degree of specificity and sensitivity (93% sensitivity and 92% specificity).

The results suggested that the VOC analyser response was not related to the parasite load in the dog peripheral blood. As the analysis gave sensitivity and specificity responses substantially better than 90%, the effect of parasite load on the VOC analyser response is unlikely to have been significant as the majority of infected animals, regardless of parasite load were detected. However, determining the limits of detection will be important in the future.

Previous work has suggested that symptomatic dogs with a greater parasite load produced greater quantities of volatiles than infected asymptomatic dogs[23]. However, asymptomatic dogs can contribute to disease transmission and VL control strategies should target infectious dogs rather than infected dogs *per se* and in particular the super-spreaders in the population[21, 45]. In this study we identified *Leishmania* DNA in circulating blood obtained by cephalic and jugular venepuncture, however the relationship between numbers of circulating parasites in peripheral blood and the infection status of the dog is unclear. In future studies, the skin parasite load, which appears to be more closely related to infectiousness[45] could be correlated with the odour profile.

In our study we used molecular techniques, PCR and qPCR, to diagnose and quantify *Le*. *infantum* infection in dogs. Although the gold standard diagnosis is considered to be the direct parasitological assessment of lymph node or bone marrow aspirates, in this study we chose to take blood and hair samples from the dogs at their homes. This methodology reduced the possibility of cross-contamination between infected and uninfected dog odour which might have occurred if the dogs had been kept together e.g. at the CCZ facility. This less invasive sampling protocol also reduced stress on the dogs, did not require large facilities (e.g. for sedation required to obtain bone marrow aspirates), reduced the risk of infection to the animal and was more likely to receive owner consent and compliance.

A recent study[46] evaluated the accuracy of serological tests, immunochromatographic (Dual Path Platform: DPP) and enzyme-linked immunosorbent (ELISA EIE), for CVL in relation to the detection of Leishmania DNA through real-time PCR) in samples from symptomatic and asymptomatic dogs. The PCR analysis demonstrated greater homogeneity between symptomatic and asymptomatic groups of infected dogs compared with DPP and ELISA. Solcà et al. showed that The diagnosis of CVL through the amplification of kinetoplast DNA presented the highest rates of sensitivity and specificity in comparison with parasitological and serological methods[47]. These authors concluded that molecular methods are required to confirm the infection. Even though serological tests are routinely employed for diagnosing CVL, they have limitations in sensitivity, especially in asymptomatic dogs, and therefore may underestimate *Leishmania* infection rates[48]. Despite the high specificity, the serological tests present low capacity to detect Leishmania infection in relation to molecular tests [49]. The authors of that study concluded that their study “demonstrated that real-time PCR identified the presence of Leishmania DNA in asymptomatic dogs that had a negative result in serological tests recommended by the official Brazilian protocol for CVL. In addition in a recent study[50], 34 out of 36 (96%) *Leishmania* isolates from dogs sampled in GV were found to be *Le*. *infantum*, the other 2 isolates were from the *Leishmania mexicana* complex, *Le*. (*Le*.) *amazonensis Le*. (*Le*.) *mexicana*). Therefore, for the purposes of the current study our molecular diagnosis was likely to be representative of the true infection status of the dogs.

The prevalence of CVL recorded in our 2017 sample (6%) is low compared to the prevalence recorded in our 2018 sample (25.6%). It is possible that the extensive monitoring carried out by Governador Valadares health authorities in the Altinópolis district of GV, where the study was carried out, immediately prior to our sample collection in 2017, had an impact on CVL prevalence.

However, these values are within the range of prevalence seen previously in studies carried out in the State of Minas Gerais generally e.g. a prevalence of 8.1% was observed in Belo Horizonte in dogs surveyed between 2008–2010[42] and 13.6% in Divinópolis in 2011[51]. In GV specifically, a study carried out in Altinópolis, between 2008–2011 found 33.8% of dogs surveyed to be infected[30] whereas a survey of dogs carried out in 2014–2015 found 22% of dogs to be positive by serology[52].

It has been suggested that change in odour of dogs infected with *Le*. *infantum* might be related to the immune response[23]. Changes in odour profile have been observed in other disease states where changes in relatively low molecular weight compounds were expressed as distinct and immediate changes arising from pathophysiological processes occurring and altering the body’s metabolism[19]. However, although the very low parasite loads in some dogs might suggest recent infection, parasite load in the peripheral blood is not indicative[53] and as this study did not determine if the dogs had seroconverted it therefore remains unclear if the odour changes are related to the host immune response or not.

Our results also suggest that there was no relationship between clinical state of infection (symptomatic, oligosymptomatic and asymptomatic) and detector response. The analyser could accurately detect asymptomatic dogs with low parasite levels as well as symptomatic dogs with high parasite loads.

It has been proposed that manipulation of the hosts chemical communication system could enhance the transmission of the parasite to the insect vector and potentially have a significant effect on the epidemiology of the disease[20, 54, 55]. Our study examined volatile odours present on the dog hair only, it did not consider the effect of other volatiles, semi-volatiles and non-volatiles from other sources e.g. breath compounds, specialized scent gland secretions, sweat, urine or faeces[56]. The source of the odours that were detected by the VOC analyser is not clear, they could have arisen from the skin, as a result of the metabolic activity of skin microbiota[57], the immune response or potentially directly from the *Le*. *infantum* parasites.

Our study did not examine the effect of other infections and the ability of the VOC analyser to differentiate between dogs infected with *Le*. *infantum* and other *Leishmania spp*. or other infections was not determined. In Governador Valadares dogs infected with *Le*. *amazonensis* have been found[50] and the sand fly vector *Lu*. *longipalpis* infected with multiple *Leishmania spp*. have been also been found[58] indicating that the epidemiological features require further work.

The application of VOC analyser technology is potentially a significant step towards the application of volatile odour analysis in diagnosis of parasitic disease. It raises the possibility that in the future a modified VOC device could provide a rapid, accurate, non-invasive point-of-care diagnostic tool for the specific diagnosis of leishmaniasis in dogs and humans. In our study we found that a small proportion of the sensor variables (2 out of 88 in 2017 and 3 out of 88 in 2018) contributed significantly to the outcome. Therefore, there is considerable scope for enhancing the sensitivity and specificity of the device through modifications to the sensor chemistry as well as incorporating further improvements to the field collection and analysis of odour. As well as further developments in robustness, portability and simplicity of the device all of which would improve the reliability and utility in the field.

A reliable, rapid, accurate, non-invasive additional point-of-care test that identifies *Leishmania* infection using a different set of disease markers in addition to the DPP CVL test could potentially eliminate the need for the in-laboratory ELISA confirmatory test that currently fails to rapidly diagnose and remove infected dogs from the population. The ability of a VOC analyser, that conforms to the WHO ASSURED criteria, to recognise infection in asymptomatic dogs and dogs with low levels of infection would be of great benefit to VL control programs.

The possible integration of a rapid, non-invasive and point-of-care test would be much better accepted by dog owners and could be an important epidemiological tool. In addition to being useful for the selection of infected animals for euthanasia, it could also be useful in the implementation, monitoring and evaluation of leishmania control activities such as insecticide impregnated dog collars that are currently being implemented and sex pheromone-based *Lu*. *longipalpis* control programs that are currently being evaluated[59, 60].

Further work to compare the sensitivity and specificity of a VOC test combined with DPP CVL diagnostics against DPP CVL combined with ELISA is required. The development of a non-invasive POC diagnostic tool based on host odour opens up a myriad range of opportunities to diagnose *Leishmania* infections in humans and other diseases such as malaria, trypanosomiasis and Chaga’s disease.

## Supporting information

S1 Table Outcome of the mixture model analysis showing the top three models for the 2017 uninfected and infected dog data. (DOCX)

S2 Table Outcome of the mixture model analysis showing the top three models for the 2018 uninfected and infected dog data. (DOCX)

S3 Table Relative importance of different sensor variables in the contribution to the clustering observed in 2017 data. (DOCX)

S4 Table Relative importance of different sensor variables in the contribution to the clustering observed in 2018 data. (DOCX)

S1 Material Rationale for the data analysis. (DOCX)
