# Supplementary material for: eNose analysis of volatile chemicals from dogs naturally infected with Leishmania infantum in Brazil
Source: PLoS Negl Trop Dis. 2019 Aug 6;13(8):e0007599. doi: 10.1371/journal.pntd.0007599 (PMC6697360; doi:10.1371/journal.pntd.0007599)
Supplement: S1 Table — (DOCX) [file pntd.0007599.s001.docx]

S1 Table.

Outcome of the mixture model analysis showing the top three models for the 2017 uninfected and infected dog data.

| Classes | Model 1 | Model 2 | Model 3 |
| --- | --- | --- | --- |
| Uninfected | EEE 1 class (-90584) | EEV 1 class (-90584) | EVE 1 class (-90584) |
| Infected | VVI 3 classes (-25125) | VEI 4 classes (-25169) | VEI 3 classes (-25180) |

Bayesian Information Criterion (BIC) values are shown within brackets. The closer the BIC value is to zero, the stronger the evidence for the model.
