# Supplementary material for: eNose analysis of volatile chemicals from dogs naturally infected with Leishmania infantum in Brazil
Source: PLoS Negl Trop Dis. 2019 Aug 6;13(8):e0007599. doi: 10.1371/journal.pntd.0007599 (PMC6697360; doi:10.1371/journal.pntd.0007599)
Supplement: S3 Table — (DOCX) [file pntd.0007599.s003.docx]

S3 Table

Relative Importance of different sensor variables in the contribution to the clustering observed in 2017 data.

| variable | *P*-value | variable | *P*-value | variable | *P*-value | variable | *P*-value |
| --- | --- | --- | --- | --- | --- | --- | --- |
| F3.Abs.5 | 0.93 | F2.Area.12 | 0.03 | F3.Abs.23 | 0.01 | F2.Area.8 | 0 |
| F1.Div.20 | 0.92 | F1.Div.12 | 0.03 | F1.Div.10 | 0.01 | F3.Abs.11 | 0 |
| F4.Des.8 | 0.07 | F2.Area.19 | 0.03 | F4.Des.4 | 0.01 | F3.Abs.22 | 0 |
| F1.Div.5 | 0.06 | F2.Area.15 | 0.03 | F2.Area.22 | 0.01 | F3.Abs.4 | 0 |
| F1.Div.19 | 0.06 | F4.Des.23 | 0.02 | F4.Des.22 | 0.01 | F2.Area.24 | 0 |
| F1.Div.13 | 0.05 | F1.Div.18 | 0.02 | F2.Area.17 | 0.01 | F4.Des.5 | 0 |
| F1.Div.16 | 0.05 | F3.Abs.24 | 0.02 | F4.Des.12 | 0.01 | F4.Des.1 | 0 |
| F1.Div.11 | 0.05 | F1.Div.7 | 0.02 | F4.Des.19 | 0.01 | F4.Des.6 | 0 |
| F4.Des.24 | 0.05 | F4.Des.16 | 0.02 | F3.Abs.17 | 0.01 | F4.Des.21 | 0 |
| F1.Div.15 | 0.05 | F3.Abs.13 | 0.02 | F4.Des.9 | 0.01 | F2.Area.16 | 0 |
| F1.Div.21 | 0.04 | F3.Abs.1 | 0.02 | F4.Des.17 | 0.01 | F3.Abs.19 | 0 |
| F2.Area.10 | 0.04 | F4.Des.10 | 0.02 | F1.Div.3 | 0.01 | F4.Des.13 | 0 |
| F2.Area.5 | 0.04 | F2.Area.20 | 0.02 | F2.Area.1 | 0.01 | F2.Area.23 | 0 |
| F3.Abs.9 | 0.03 | F1.Div.6 | 0.02 | F2.Area.3 | 0.01 | F2.Area.21 | 0 |
| F3.Abs.7 | 0.03 | F1.Div.17 | 0.02 | F4.Des.3 | 0.01 | F2.Area.7 | 0 |
| F1.Div.8 | 0.03 | F4.Des.20 | 0.02 | F1.Div.9 | 0 | F4.Des.7 | 0 |
| F4.Des.11 | 0.03 | F2.Area.6 | 0.02 | F3.Abs.21 | 0 | F1.Div.1 | 0 |
| F1.Div.24 | 0.03 | F2.Area.9 | 0.02 | F3.Abs.6 | 0 | F2.Area.4 | 0 |
| F3.Abs.18 | 0.03 | F3.Abs.15 | 0.02 | F3.Abs.12 | 0 | F2.Area.18 | 0 |
| F1.Div.4 | 0.03 | F3.Abs.10 | 0.01 | F3.Abs.16 | 0 | F2.Area.13 | 0 |
| F2.Area.11 | 0.03 | F1.Div.23 | 0.01 | F3.Abs.8 | 0 | F3.Abs.3 | 0 |
| F1.Div.22 | 0.03 | F3.Abs.20 | 0.01 | F4.Des.18 | 0 | F4.Des.15 | 0 |

Abs = absorbance, Des = desorbance, Area = area under the curve, Div = divergence.
